# Probing the Effects of Pyrimidine Functional Group Switches on Acyclic Fleximer Analogues for Antiviral Activity

**DOI:** 10.3390/molecules24173184

**Published:** 2019-09-02

**Authors:** Mary K. Yates, Payel Chatterjee, Mike Flint, Yafet Arefeayne, Damjan Makuc, Janez Plavec, Christina F. Spiropoulou, Katherine L. Seley-Radtke

**Affiliations:** 1 Department of Chemistry and Biochemistry, University of Maryland, Baltimore County, Baltimore, MD 21250, USA; 2 Viral Special Pathogens Branch, Centers for Disease Control and Prevention, Atlanta, GA 30329, USA; 3 Slovenian NMR Center, National Institute of Chemistry, Hajdrihova 19, SI-1000 Ljubljana, Slovenia

**Keywords:** nucleoside, SAR, filovirus, flavivirus, fleximers

## Abstract

Due to their ability to inhibit viral DNA or RNA replication, nucleoside analogues have been used for decades as potent antiviral therapeutics. However, one of the major limitations of nucleoside analogues is the development of antiviral resistance. In that regard, flexible nucleoside analogues known as “fleximers” have garnered attention over the years due to their ability to survey different amino acids in enzyme binding sites, thus overcoming the potential development of antiviral resistance. Acyclic fleximers have previously demonstrated antiviral activity against numerous viruses including Middle East Respiratory Syndrome coronavirus (MERS-CoV), Ebola virus (EBOV), and, most recently, flaviviruses such as Dengue (DENV) and Yellow Fever Virus (YFV). Due to these interesting results, a Structure Activity Relationship (SAR) study was pursued in order to analyze the effect of the pyrimidine functional group and acyl protecting group on antiviral activity, cytotoxicity, and conformation. The results of those studies are presented herein.

## 1. Introduction

**Figure 1:**
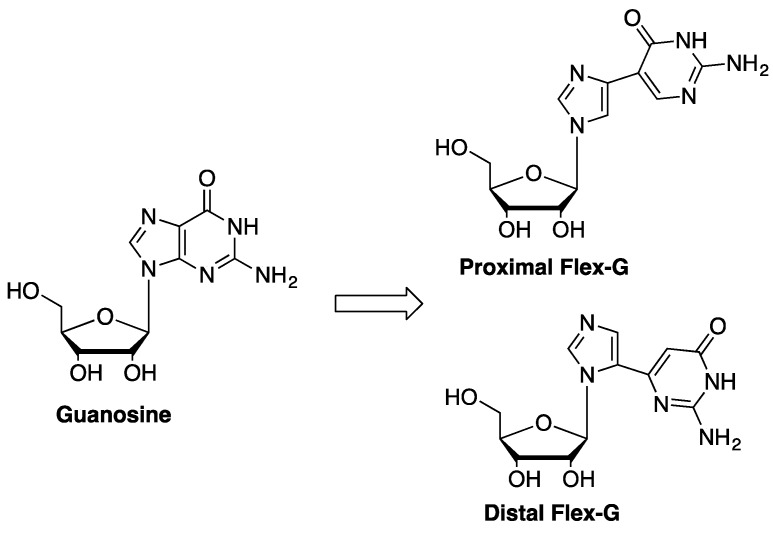
Structure of guanosine compared to the corresponding proximal and distal fleximer analogues.

Nucleoside analogues have long served as the cornerstone for antiviral therapeutics due to their ability to inhibit viral DNA or RNA replication [1,2]. By altering different components of the nucleoside, such as the nucleobase, sugar moiety, and phosphate group, medicinal chemists can develop novel analogues for their use in various therapeutics [1,2]. Over the past decade, research in the Seley-Radtke lab has focused on the development of various types of flexible nucleoside analogues, called “fleximers”, that have demonstrated the ability to overcome point mutations within the binding site of biologically significant enzymes (Figure 1) [3,4,5,6,7,8,9,10,11,12,13,14,15,16,17,18,19,20]. 

Fleximers feature a purine ring that is split into its imidazole and pyrimidine components, but remain connected by a single carbon–carbon bond between the C5 of the imidazole and the C6 of the pyrimidine in *distal* fleximers, and between the C4 of the imidazole and the C5 of the pyrimidine in *proximal* fleximers [3,4,7] This strategic design endows fleximers with an inherent flexibility that (1) retains hydrogen bond motifs that are necessary for enzyme recognition, (2) adapts to flexible binding site environments, and (3) allows access to different amino acids in enzyme binding sites that were previously unattainable by the more rigid parent nucleoside [6,8,21]. 

**Figure 2:**
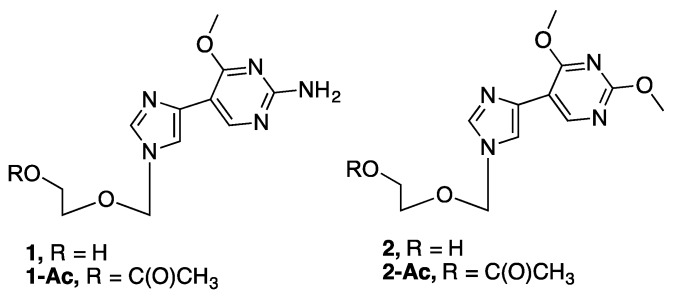
Parent fleximer analogues demonstrating broad spectrum antiviral activity [3,4,5,6,7,8,9,10,11,12,13,14,15,16,17,18,19,20].

Most of the recent studies within the Seley-Radtke lab have focused on the development of fleximer analogues for use in antiviral and anticancer therapeutics [11,17,20]. In particular, fleximer analogues based on the structure of the Food and Drug Administration (FDA)-approved drug Acyclovir have demonstrated great promise, with compound 1 demonstrating low micromolar activity against coronaviruses [17], filoviruses [20], and most recently, flaviviruses (Figure 2, unpublished results).

**Figure 3:**
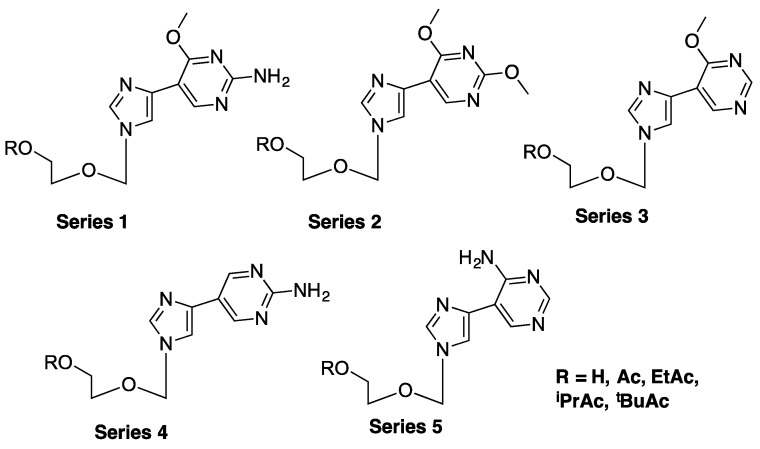
Target analogues for the SAR study.

In parallel, a secondary series featuring a methoxy at the 2-position on the pyrimidine was developed in order to better analyze the effect of hydrogen bonding motifs on antiviral activity. This series was also tested against the aforementioned viruses; however, these analogues were not more active than compound **1**. Furthermore, the addition of an acetate protecting group (**1-Ac**) led to a decrease in antiviral activity, but also a large decrease in cytotoxicity (unpublished results). In order to explore the effects of different hydrogen bond donor and acceptor groups on the fleximer pyrimidine ring, as well as the effect of various acetate protecting groups on antiviral activity, an SAR study was pursued. Herein, we report the synthesis and antiviral evaluation of series **1**–**5** (Figure 3).

## 2. Results

### Chemistry

**Scheme 1:**
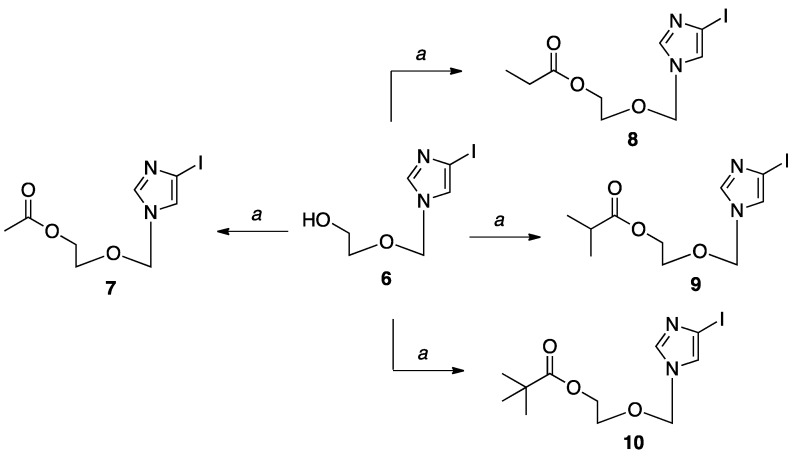
Reagents and conditions: a. Acetic anhydride (for **7**), propionic anhydride (for **8**), isobutyric anhydride (for **9**), or trimethylacetic anhydride (for **10**), triethylamine (TEA), DIPEA, anh. CH_2_Cl_2_, rt, 3h.

Synthesis of these analogues began with modifying the acyclic sugar moiety **6** by the addition of different acyl protecting groups (Scheme 1). The synthesis of **6** and **7** was previously published by our group [17,20], and all intermediates were obtained in a similar fashion, with the addition of the appropriate anhydride followed by triethylamine and diisopropylamine (DIPEA) in anh. CH_2_Cl_2_ to give **8–10** as clear to pale yellow oils in high yields (78–99%). 

**Scheme 2:**
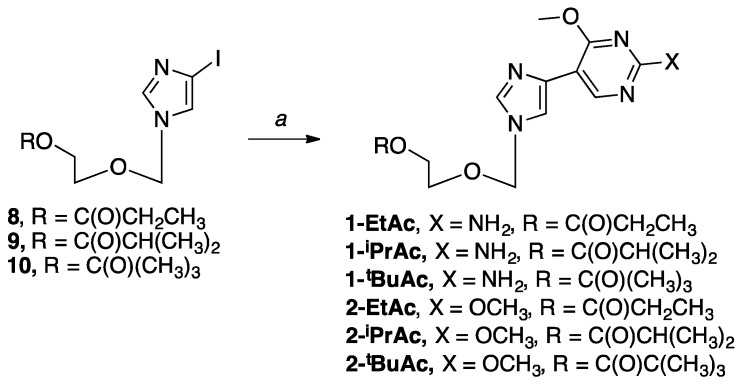
Reagents and conditions: a. 4-methoxy-5-(tributylstannyl)pyrimidin-2-amine (for series 1) or 2,4-dimethoxy-5-(tributylstannyl)pyrimidine (for series 2), Pd(PPh3)4, CuI, CsF, anh. dimethylformamide (DMF), 65 °C, 3 h.

As the synthesis of **1, 1-Ac, 2,** and **2-Ac** have already been reported [17,20], the remaining analogues in these series were pursued first. Using the appropriate stannyl pyrimidine under Stile conditions, **1- EtAc, 1-^i^PrAc, 1-^t^BuAc, 2-EtAc, 2-^i^PrAc,** and **2-^t^BuAc** were all realized in good yields (Scheme 2).

**Scheme 3:**
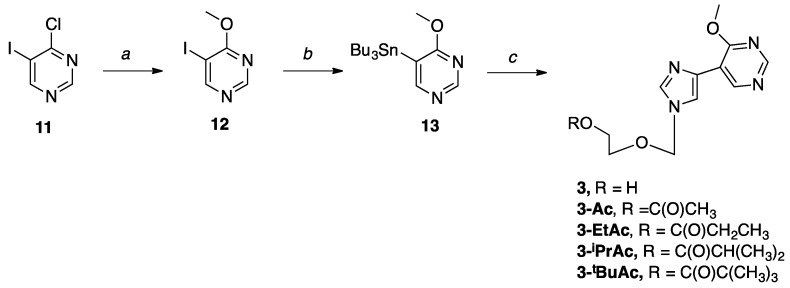
Reagents and conditions: a. NaOMe (30% by weight in CH_3_OH), CH_3_OH, rt, 6h; b. (SnBu_3_)_2_, Pd_2_dba_3_•CHCl_3_, anhydrous (anh.) DMF, 65 °C, 2.5 h; c. **6** (for **3**), **7** (for **3-Ac**), **8** (for **3-EtAc**), **9** (for **3-^i^PrAc**), or **10** (for **3-^t^BuAc**), Pd(PPh_3_)_4_, CuI, CsF, anh. DMF, 65 °C, 3 h.

After the successful completion of series **1** and **2**, attention turned toward the synthesis of the 4-methoxypyrimidine series **3** (Scheme 3). Synthesis began with commercially available 4-chloro-5-iodopyrimidine (**11**), where the addition of sodium methoxide (30% by weight in CH_3_OH) gave 5-iodo-4-methoxypyrimidine **12** in good yields. Following purification, the stannyl pyrimidine **13** was realized with bistributyltin, the tris(dibenzylideneacetone)dipalladium catalyst, and mild heating. 

Coupling of the various sugars with the stannyl pyrimidine **12** was then accomplished using Stille palladium catalyzed cross-coupling methods to give analogues **3, 3-Ac, 3-EtAc, 3-^i^PrAc,** and **3-^t^BuAc** in good yields (30–45%).

**Scheme 4:**
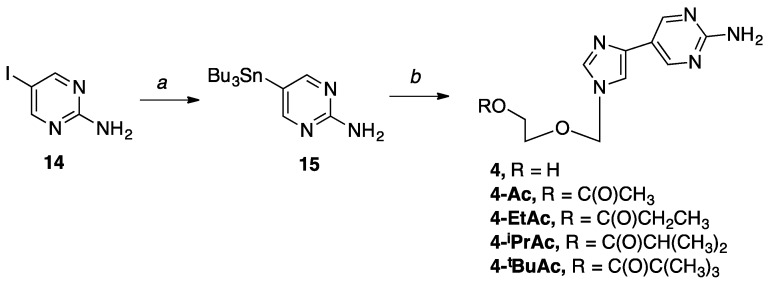
Reagents and conditions: a. (SnBu_3_)_2_, Pd_2_dba_3_•CHCl_3_, anh. DMF, 65 °C, 2.5 h; b. **6** (for **4**), **7** (for **4-Ac**), **8** (for **4-EtAc**), **9** (for **4-^i^PrAc**), or **10** (for **4-^t^BuAc**), Pd(PPh_3_)_4_, CuI, CsF, anh. DMF, 65 °C, 3 h.

Once the 4-methoxypyrimidine series **3** was successfully synthesized, the 2-aminopyrimidine series **4** was pursued. Similar to the previous series, synthesis of series **5** started with commercially available 2-amino-5-iodopyrimidine (**14**) and bis(tributyltin) to give the stannyl pyrimidine **15** in good yields (86%, Scheme 4). Following purification of the stannyl pyrimidine, Stille palladium catalyzed cross-coupling methods were employed to realize compounds **4** through **4-^t^BuAc** in high yields compared to the previous series (>50% for all analogues compared to an average of 15% for series **1**). 

**Scheme 5:**
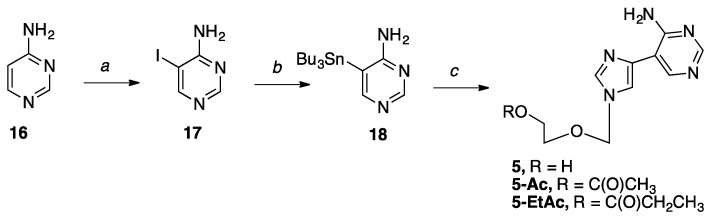
Reagents and conditions: a. N-iodosuccinimide (NIS), AcOH, 85 °C, 3 h; b. (SnBu_3_)_2_, Pd_2_dba_3_•CHCl_3_, DMF, 65 °C, 2.5 h; c. **6** (for **5**), **7** (for **5-Ac**), or **8** (for **5-EtAc**)**,** CsF, CuI, PPh_3_, Pd_2_dba_3_•CHCl_3_, DMF, 65 °C, 3–5 h.

Finally, the 4-aminopyrimidine series **5** was originally pursued using similar methods, starting with commercially available 4-aminopyrimidine (**16**, Scheme 5). 

Using N-iodosuccinimide in glacial acetic acid, 4-amino-5-iodopyrimidine **17** was realized, followed by the addition of bis(tributyltin) to give the stannyl coupling partner **18**. Subsequent coupling with **6**, **7**, and **8** gave very low yields (<10%) and gave rise to purifications issues, as it was difficult to isolate the pure product without residual tin impurities. Products **5** to **5-EtAc** were also fairly soluble in water; thus, using a 1-M potassium fluoride (KF) wash to convert the soluble tin impurity to the insoluble SnBu_3_F was unsuccessful. Furthermore, the addition of the acyl protecting group had no effect on tin impurity removal, thus the deprotection of **5-Ac** to give **5** with methanolic ammonia was ineffective as well. As such, a protecting group for the 4-aminopyrimidine was envisioned in attempts to make purification more facile and increase the overall yields.

**Scheme 6:**
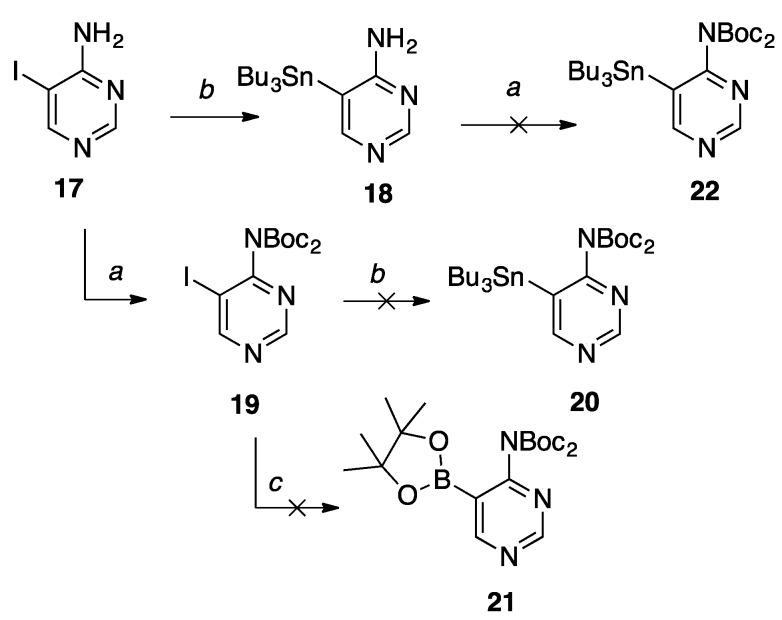
Reagents and conditions: a. Boc_2_O, dimethylaminopyridine (DMAP), dichloromethane (DCM), rt, 2h; b. (SnBu_3_)_2_, Pd_2_dba_3_•CHCl_3_, DMF, 65 °C, 3 h; c. bis(pinacolato)diboron, KOAc, PdCl_2_(dppf) CHCl_3_, DMF, 120 °C.

First, protection of the 4-amino group was tried using two equivalents of di-tert-butyl dicarbonate to give a di-boc protected amine, which could easily be deprotected after coupling to the sugar using mild acidic conditions (Scheme 6). 

**Scheme 7:**
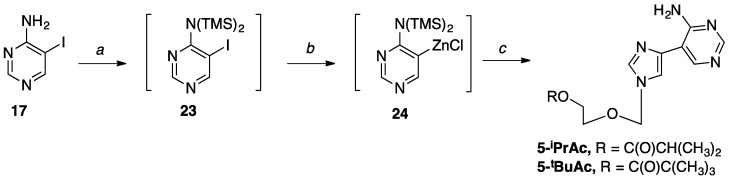
Reagents and conditions: a. EtMgBr, trimethylsilyl chloride (TMSCl), tetrahydrofuran (THF), −78 °C; b. EtMgBr, ZnCl_2_, THF, −78°C – rt, 2 h; c. **9** (for **5-^i^PrAc**), or **10** (for **5-^t^BuAc**), Pd(PPh_3_)_4_, CuI, THF, rt, 18 h.

Protection of the 5-iodo intermediate **17** using di-tert-butyl dicarbonate was successful in producing the di-boc compound **19**; however, subsequent attempts at installing the stannyl component (**20**) were unsuccessful. Furthermore, attempts at installing the boronic ester (**21**) for use in more mild Suzuki coupling conditions were also unsuccessful. Final attempts at employing the di-boc protecting group tried protecting the stannyl pyrimidine **18**; however, this method also proved ineffective. As such, attention turned toward using an in situ protection of the amine group followed by Negishi conditions for the **5-^i^PrAc** and **5-^t^BuAc** compounds, as these conditions had previously been successful for other fleximers developed in our lab (Scheme 7) [22].

Compound **17** was first protected in situ with chlorotrimethylsilane to give intermediate **23**, which was subsequently complexed with ZnCl_2_ to give intermediate **24**. Dropwise addition of the zinc complex to either **9** or **10** gave **5-^i^PrAc** and **5-^t^BuAc**, respectively. While the coupling itself went well, later purification of these analogues proved difficult. However, the addition of 10% NH_4_OH to CH_3_OH in the chromatography gradient greatly decreased streaking and allowed for purification of these products (yields ~48%).

## 3. Discussion

As the different series were completed, they were sent for testing to collaborators at the Centers for Disease Control (CDC) for testing against EBOV. Briefly, a recombinant reporter virus, EBOV-Makona expressing ZsGreen (rEBOV/ZsG), was used to infect compound-treated Huh7 cells at a multiplicity of infection (MOI) of 0.3. Three days later, ZsGreen fluorescence was measured, and EC50 values were determined. Concurrently, the compound effects on cell viability were determined by measuring the cellular ATP content of compound-treated, mock-infected cells [20,23,24]. The results of this study are summarized in Table 1.

None of the analogues tested demonstrated any cytotoxicity up to 100 µM, and while compound **1** remained the most active against EBOV, interesting trends emerged regarding the acyl protecting groups as well as the moieties on the pyridmine ring. 

When the parent compounds of each series were tested, it was found that changing the hydrogen bond-donating amino group at the 2-poistion (compound **1**) to a hydrogen bond-accepting methoxy group (compound **2**) decreased activity (1.49 µM compared to 23.44 µM, *p* = 0.0082). Furthermore, when there is a hydrogen atom at the 2-position (compound **3**), a decrease in activity compared to compound **1** was also observed (1.49 µM compared to 78.88 µM, *p* = 0.0061). Compounds **4** and **5**, which feature only an amino group in the 2-position and 4-position, respectively, did not display any activity against EBOV. Interestingly, activity could be recovered for series **4** and **5,** as well as improved for series **3,** by altering the acyl protecting group. For instance, the addition of the acetate protecting group with **3-Ac** greatly increased activity compared to the parent analogue **3** (35.36 µM compared to 78.88 µM, *p* = 0.0162). However, the addition of the acyl protecting group seemed to affect each series differently, as compound **1-Ac** was not active against EBOV. In contrast, the parent compound **1** was very active, but **4-Ac** was active against EBOV (EC_50_ = 52.89 µM), whereas the parent compound **4** was not active. For series **2,** the activity of **2-Ac** appeared to increase, but did not reach statistical significance (*p* = 0.7922). 

For series **2** and **4**, there was a loss in activity with the ethyl acyl (EtAc) analogues compared to analogues with only an acetate group, such as **2-Ac** compared to **2-EtAc** as well as **4-Ac** compared to **4-EtAc**. However, activity returned with the isopropyl acyl (^i^PrAc) protected compounds. Finally, the *tert*-butyl acyl (^t^BuAc) protected analogues were variable in activity across the series, though for the most part, these analogues were not significantly different in activity from the acetate (Ac). For example, **3-Ac** demonstrated activity to 35.36 µM, **3-^t^BuAc** demonstrated activity to 51.40 µM (*p* = 0.1709), and **4-Ac** demonstrated activity to 52.89 µM compared to **4-^t^BuAc** at 34.42 µM (*p* = 0.1949). The adenosine series **5** were not active except for the ^t^BuAc, which demonstrated weak activity. These results, along with the potent activity observed for compound **1**, which also bears a 4-methoxy group, suggests that the 4-methoxy group is an important determinant for antiviral activity.

The difference in activity between the Ac, EtAc, and ^i^PrAc is an interesting trend, as the EtAc analogues were consistently the least active analogues. In order to analyze if this trend was due to a change in lipophilicity or due to branching, the logP values were determined for each analogue (Table 2).

LogP values are a common measure of lipophilicity, and the greater the value, the more lipophilic the compound. Not surprisingly, all the parent analogues (R group = H) were the least lipophilic of the series, with the amino series **4** and **5** demonstrating the lowest lipophillicity. On average, the difference between the parent analogues and the acetate analogues (R = Ac) was not substantial; however, the ethyl acyl analogues (R = EtAc) were substantially more lipophilic than the acetate analogues (for example, −0.14 compared to 0.52 for series **5**). Not surprisingly, the increased branching also led to an increase in lipophilicity, as the ^t^BuAc analogues were the most lipophilic of their series. 

While increased lipophillicity is typically associated with a better drug candidate, there is a limit where increased lipophillicity affects potency. For instance, the isopropyl acyl analogue **2-^i^PrAc** demonstrated an EC_50_ of 31.96 ± 9.12 µM against EBOV and a logP of 2.15, whereas **2-^t^BuAc** has an EC_50_ of 81.34 ± 10.15 µM and a logP of 2.85. Furthermore, the isopropyl acyl analogue **3-^i^PrAc** demonstrates an EC_50_ of 38.29 ± 28.15 µM and a logP of 1.76, whereas **3-^t^BuAc** has an EC_50_ of 51.40 ± 10.01 µM and a logP of 2.47. This suggests that compounds with logP values over 2.8 are too lipophilic, and thus are less active against EBOV. Interestingly, compound **4-^i^PrAc** is less active than the more branched **4-^t^BuAc** and is less lipophilic (logP = 0.88 vs 1.59), suggesting that compound with logP values greater than 1 may be more active. All together, these results suggest that lipophillicity and not branching affects antiviral activity.

Previous studies with the Seley-Radtke lab found that in solution, ribose fleximers preferred the typical anti-conformation found with nucleosides [21]. As such, studies were pursued in order to determine if the acyclic fleximer analogues also preferred anti-conformation in solution. ^1^H-NMR spectra were recorded on 300-MHz NMR spectrometer at 3-mM concentration in 1% dimethylsulfoxide (DMSO)-*d*_6_/D_2_O. 

**Figure 4:**
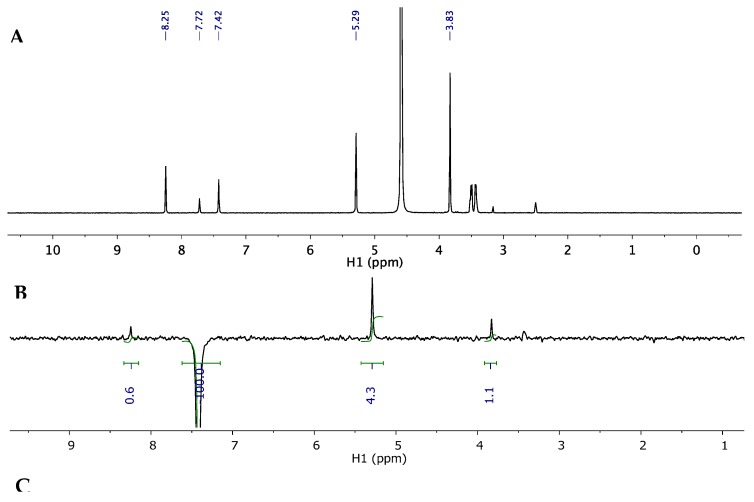
(**A**) ^1^H-NMR spectrum of **1** in 1% DMSO/D_2_O at 25 °C; 1D NOESY Nuclear Magnetic Resonance (NMR) spectra of **1** in 1% DMSO/D_2_O at 25 °C; selective inversion of (**B**) H11 and (**C**) H6 proton; mixing time 1.5 s.

Using well-resolved NMR signals of H6, H8, and H11 aromatic protons (Figure 4A), an assessment of the conformational preferences along the C11–C10–C5–C6 torsion angle was possible through the use of 1D Nuclear Overhauser Effect Spectroscopy (NOESY) experiments.

Selective inversion of the H11 proton resulted in strong NOE enhancement of 4.3% at H1’, whereas other NOE enhancements were relatively weak (Figure 4B). The lack of strong NOE enhancements between H11 and H6 suggests that anti-orientation along the C11–C10–C5–C6 torsion angle is the predominant conformation of compound **1** in solution. Additionally, inversion of the H6 proton showed no NOE enhancements (Figure 4C), which confirms that H6 and H11 are spatially apart, overall supporting anti-conformation.

**Figure 5:**
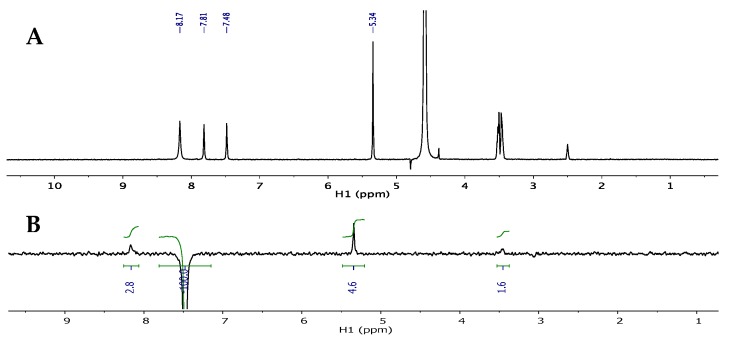
(**A**) ^1^H-NMR spectrum of **5** in 1% DMSO/D_2_O at 25 °C. (**B**) 1D NOESY NMR spectra of **5** in 1% DMSO/D_2_O at 25 °C; selective inversion of H11; mixing time 1.5 s.

These studies also found that compounds **1-Ac, 2,** and **2-Ac** also favored anti-conformation along the C11–C10–C5–C6 torsion angle. Interestingly, selective inversion of the H11 proton of compound **5** resulted in relatively strong NOE enhancement of 4.6% at H1’, as well as moderate NOE enhancement of 2.8% at H6 (Figure 5B). These results suggest that *syn* orientation along the C11–C10–C5–C6 torsion angle is predominant. This conformation was also determined to be prominent in the acetate prodrug **5-Ac**. 

**Figure 6:**
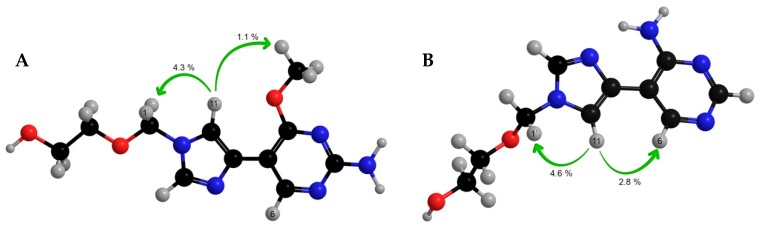
(**A**) Anti-conformation of compound **1** compared to (**B**) syn conformation of compound **5**.

These results are particularly interesting due to the lack of antiviral activity seen with series **5** against EBOV. The difference in the conformations between compounds **1, 2**, and **5** may offer a reason for the drastic differences in antiviral activity observed. The differences in orientation are particularly highlighted in Figure 6. 

While compounds **1, 1-Ac, 2, 2-Ac, 5,** and **5-Ac** have been analyzed in these studies, it will be interesting to see if the 4-methoxy series **3** or the 2-amino series **4** also demonstrate different orientations compared to series **1** or **5.** Due to the antiviral activity found with series **3** and **4**, it is expected that these analogues also are predominately found in the anti-conformation.

## 4. Materials and Methods

### 4.1. Chemistry General Information

All reactions were performed using oven-dried glassware under a nitrogen atmosphere with magnetic stirring. Reagents were purchased from Sigma-Aldrich (Rockville, MD, USA), Alfa Aesar (Haverhill, MA, USA), and Combiblocks (San Diego, CA, USA). Solvents were either purchased as anhydrous or were dried using the MBRAUN solvent purification system (MB-SPS) (MBRAUN, Stratham, NH, USA). Reactions were monitored by thin layer chromatography (TLC) using EMD silica gel 60 F254 coated glass-backed TLC plates (Milliporesigma, Rockville, MD, USA) and visualized with a UV lamp and/or KMnO_4_ stain (Fisher Scientific, Pittsburgh, PA, USA). Column chromatography was performed on a CombiFlash^®^Rf automated chromatography using REdiSep Rf silica (Teledyne Isco, Lincoln, NE, USA). High-Pressure Liquid Chromatography (HPLC) purification was performed on an LC-8A Shimadzu preparation liquid chromatograph (Shimadzu, Columbia, MD, USA) using a Zorbax SB C18 column 9.4 × 250 mm, 5 µm. Samples were lyophilized utilizing a Labconco Freezone 1 L lyophilizer. ^1^H-NMR spectra was recorded on a JEOL Eclipse ECX (400 MHz) (JEOL USA, Inc., Peabody, MA, USA) or a Bruker Avance III HD (500 MHz) spectrometer (Bruker BioSpin Corporation, Billerica, MA, USA) Chemical shifts are reported in parts per million (δ ppm) from tetramethylsilane with the solvent resonance as an internal standard. Data for the ^1^H-NMR are reported as follows: br = broad, s = singlet, d = doublet, t = triplet, q = quartet, dd = doublet of doublets, m = multiplet. ^13^C-NMR were recorded on a JOEL Eclipse ECX (126 MHz) or a Bruker Advance III HD (101 MHz) spectrometer with complete proton decoupling. Data for ^13^C-NMR are reported in terms of chemical shifts (δ ppm) with the solvent resonance as the internal standard. Mass spectrometry was performed on a Bruker AmaZon X quadrupole ion trap mass spectrometer using electrospray ionization (ESI) (Bruker BioSpin Corporation, Billerica, MA, USA). The purity of all compounds tested in biological assays were verified by elemental analyses performed by Atlantic Microlabs (Atlantic Microlabs, Norcross, GA, USA). LogP values were calculated using the ChemDraw program by PerkinElmer Informatics (PerkinElmer Informatics, Cambridge, MA, USA).

### 4.2. Synthesis

*General procedure for the acyl-protected sugars***8**–**10**. To a solution of **6** (1.5 g, 5.96 mmol) in anh. CH_2_Cl_2_ (100 mL) under a nitrogen balloon was added the corresponding anhydride (2 equivalents), triethylamine (2.50 mL, 17.85 mmol), and DMAP (0.18 g, 1.49 mmol) with stirring at room temperature. The reaction stirred for 3 hours at room temperature; then, the solvent was removed in vacuo. Purification by flash column chromatography on silica gave the products in good yields.

*Characterization of 2-((4-iodo-1H-imidazol-1-yl)methoxy)ethyl propionate* (**8**). The anhydride used was propionic anhydride (1.52 mL, 11.90 mmol). The crude mixture was purified by flash column chromatography on silica (0–100% EtOAc in hexanes) to give the product as a clear oil (1.78 g, 99%); R_f_ = 0.42 (1:1 EtOAc: Hex); ^1^H-NMR (500 MHz, CDCl_3_) δ 7.41 (s, 1H), 7.05 (s, 1H), 5.19 (s, 2H), 4.01–4.03 (t, *J* = 4.7 Hz, 2H), 3.47–3.49 (t, *J* = 4.6 Hz, 2H), 2.14–2.19 (m, 2H), 0.93–0.96 (t, *J* = 7.6 Hz, 3H); ^13^C-NMR (126 MHz, CDCl_3_) δ 174.43, 139.03, 124.30, 83.12, 76.41, 66.72, 62.80, 27.58, 9.13; MS (ESI+) *m/z* calcd for C_9_H_13_IN_2_O_3_ [M + H]^+^ 325.00 found: 325.01.

*Characterization of 2-((4-iodo-1H-imidazol-1-yl)methoxy)ethyl isobutyrate* (**9**). The anhydride used was isobutyric anhydride (1.98 mL, 11.90 mmol). The crude mixture was purified by flash column chromatography on silica (0–100% EtOAc in hexanes) to give the product as a clear oil (2.0 g, 99%); R_f_ = 0.53 (1:1 EtOAc: Hex;^1^H-NMR (500 MHz, CDCl_3_) δ 7.26 (s, 1H), 6.89 (s, 1H), 5.01 (s, 2H), 3.77–3.78 (m, 2H), 3.27–3.28 (m, 2H), 2.14–2.15 (m, 1H), 0.72–0.78 (d, *J* = 4.4 Hz, 6H); ^13^C-NMR (101 MHz, CDCl_3_) δ 176.45, 139.17, 124.53, 82.33, 76.12, 66.70, 62.46, 33.47, 18.79. MS (ESI+) *m/z* calcd for C_10_H_15_IN_2_O_3_ [M + Na]^+^ 361.15 found: 361.03.

*Characterization of 2-((4-iodo-1H-imidazol-1-yl)methoxy)ethyl pivalate* (**10**). The anhydride used was trimethylacetic anhydride (2.40 mL, 11.90 mmol). The crude mixture was purified by flash column chromatography on silica (0–100% EtOAc in hexanes) to give the product as a clear oil (1.55 g, 78%); R_f_ = 0.53 (1:1 EtOAc: Hex; ^1^H NMR (400 MHz, CDCl_3_) δ 7.44 (s, 1H), 7.07 (s, 1H), 5.22 (s, 2H), 4.06–4.13 (t, *J* = 4.1 Hz, 2H), 3.48–3.52 (t, *J* = 4.1 Hz, 2H), 1.07–1.15(s, 9H); ^13^C NMR (125 MHz, CDCl_3_) δ 177.42, 139.53, 130.08, 124.92, 76.02, 66.76, 62.70, 38.35, 28.88. MS (ESI+) *m/z* calcd for C_11_H_17_IN_2_O_3_ [M + Na]^+^ 375.17 found: 375.05.

*General procedure for Stille cross coupling.* The sugar (1 equivalent) and stannyl reagent (1.2 equivalents) were dried in a three-neck flash in vacuo for two hours, and then were suspended in anh. DMF (100 mL) under direct nitrogen bubbling. CsF (2 equivalents) and CuI (0.4 equivalents) were added, and the reaction was stirred for 10 min. Tetrakis (0.2 equivalents) was then added, and the reaction was heated to 65 °C and stirred with direct nitrogen bubbling for 2.5 h. After completion, the reaction was cooled, and the solvent was removed in vacuo. The crude mixture was then purified three to four times by flash column chromatography on silica to give the pure products.

*2-((4-(2-Amino-4-methoxypyrimidin-5-yl)-1H-imidazol-1-yl)methoxy)ethyl propionate (1-EtAc).* The sugar **8** (0.75 g, 2.31 mmol) and 4-methoxy-5-(tributylstannyl)pyrimidin-2-amine (1.15 g, 2.77 mmol) were used. The product was purified three times by flash column chromatography on silica (0–25% CH3OH in CH_2_Cl_2_) to give the product as a white solid (0.041 g, 9%); ^1^H NMR (500 MHz, CD_3_OD) δ 8.83 (s, 1H), 7.62 (s, 1H), 7.40 (s, 1H), 5.32 (s, 2H), 4.20–4.21 (t, *J* = 4.7 Hz, 2H), 4.01 (s, 3H), 3.62–3.64 (t, *J* = 4.7 Hz, 2H), 2.30–2.35 (m, 2H), 1.09–1.12 (t, *J* = 7.6 Hz, 3H); ^13^C NMR (126 MHz, CDCl_3_) δ 174.30, 166.23, 161.08, 154.83, 136.85, 135.41, 117.00, 105.92, 76.46, 66.58, 62.70, 53.66, 27.35, 9.00; Elemental analysis calcd for C_14_H_19_N_5_O_4_ + 0.25 CH_4_OH + 0.15 H_2_O: C, 51.55; H, 6.16; N, 21.09; Found: C, 51.58; H, 6.12, N, 21.06.

*2-((4-(2-Amino-4-methoxypyrimidin-5-yl)-1H-imidazol-1-yl)methoxy)ethyl isobutyrate (1-^i^PrAc).* The sugar **9** (0.80 g, 2.37 mmol) and 4-methoxy-5-(tributylstannyl)pyrimidin-2-amine (1.18 g, 2.84 mmol) were used. The product was purified three times by flash column chromatography on silica (0–25% CH3OH in CH_2_Cl_2_) to give the product as a pale yellow oil (0.09 g, 12%); ^1^H NMR (500 MHz, CDCl_3_) δ 8.80 (s, 1H), 7.60 (s, 1H), 7.39 (s, 1H), 5.31 (s, 2H), 4.17–4.20 (t, *J* = 5.6 Hz, 2H), 3.98 (s, 3H), 3.59–3.61 (t, *J* = 5.3 Hz, 2H), 2.48–2.55 (m, 1H), 1.10–1.12 (d, *J* = 9.2 Hz, 6H); ^13^C NMR (126 MHz, CDCl_3_) δ 176.86, 166.42, 161.37, 155.24, 137.35, 135.49, 117.03, 106.04, 76.41, 66.58, 62.66, 53.58, 33.78, 18.96; Elemental analysis calcd for C_15_H_21_N_5_O_4_ + 0.65 H_2_O: C, 51.91; H, 6.48; N, 20.18; Found: C, 51.60; H, 6.09; N, 20.18.

*2-((4-(2-Amino-4-methoxypyrimidin-5-yl)-1H-imidazol-1-yl)methoxy)ethyl pivalate (1-^t^BuAc).* The sugar **10** (0.75 g, 2.13 mmol) and 4-methoxy-5-(tributylstannyl)pyrimidin-2-amine (1.06 g, 2.56 mmol) were used. The product was purified three times by flash column chromatography on silica (0–20% CH3OH in CH_2_Cl_2_) to give the product as a clear oil (0.08 g, 11%); ^1^H NMR (500 MHz, CDCl3) δ 8.78 (s, 1H), 7.67 (s, 1H), 7.41 (s, 1H), 5.36 (s, 2H), 4.23–4.25 (t, *J* = 4.0 Hz, 2H), 4.11 (s, 3H), 3.65–3.67 (t, *J* = 4.0 Hz, 2H), 1.19–1.27 (s, 9H); ^13^C NMR (126 MHz, CDCl_3_) δ 176.48, 166.35, 153.71, 137.37, 134.27, 117.33, 104.72, 76.09, 66.45, 62.77, 52.73, 38.31, 26.05; Elemental analysis calcd for C_16_H_23_N_5_O_4_ + 0.1 CH_4_OH + 0.95 H_2_O: C, 52.31; H, 6.90; N, 18.94; Found: C, 52.41; H, 6.74; N, 18.78.

*2-((4-(2,4-Dimethoxypyrimidin-5-yl)-1H-imidazol-1-yl)methoxy)ethyl propionate (2-EtAc).* The sugar **8** (0.75 g, 2.31 mmol) and 2,4-dimethoxy-5-(tributylstannyl)pyrimidine (1.19 g, 2.78 mmol) were used. The product was purified three times by flash column chromatography on silica (50–100% EtOAc in hexanes) to give the product as a pale yellow oil (0.25 g, 36%); ^1^H NMR (500 MHz, CDCl_3_) δ 8.82 (s, 1H), 7.91 (s, 1H), 7.65 (s, 1H), 5.47 (s, 2H), 4.19–4.21 (t, *J* = 3.5 Hz, 2H), 4.14 (s, 3H), 4.02 (s, 3H), 3.71–3.72 (t, *J* = 3.6 Hz, 2H), 2.28–2.33 (m, 2H), 1.05–1.08 (t, *J* = 7.6 Hz, 3H); ^13^C NMR (126 MHz, CD_3_OD) δ 174.44, 166.98, 163.58, 154.80, 138.21, 132.99, 118.74, 108.50, 76.34, 66.67, 62.73, 54.08, 53.49, 26.74, 8.01; Elemental analysis calcd for C_15_H_20_N_4_O_5_ + 0.05 H_2_O + 0.2 CH_2_Cl_2_: C, 51.54; H, 5.83; N, 15.82; Found: C, 51.67; H, 5.64; N, 15.61.

*2-((4-(2,4-Dimethoxypyrimidin-5-yl)-1H-imidazol-1-yl)methoxy)ethyl isobutyrate (2-^i^PrAc).* The sugar **9** (0.25 g, 0.74 mmol) and 2,4-dimethoxy-5-(tributylstannyl)pyrimidine (0.38 g, 0.89 mmol) were used. The product was purified twice times by flash column chromatography on silica (25–100% EtOAc in hexanes) to give the product as a light orange oil (0.06 g, 24%); ^1^H NMR (400 MHz, CDCl_3_) δ 8.96 (s, 1H), 7.61 (s, 1H), 7.43 (s, 1H), 5.32 (s, 2H), 4.14–4.19 (t, *J* = 3.4 Hz, 2H), 4.03 (s, 3H), 3.95 (s, 3H), 3.55–3.57 (t, *J* = 3.4 Hz, 2H), 2.45–2.47 (m, 1H), 1.05–1.09 (d, *J* = 6.9 Hz, 6H); ^13^C NMR (101 MHz, CDCl_3_) δ 176.95, 166.89, 163.73, 155.65, 137.12, 134.63, 117.99, 109.21, 76.91, 66.71, 62.61, 54.86, 54.12, 33.84, 18.95; Elemental analysis calcd for C_16_H_22_N_4_O_5_: C, 54.85; H, 6.33; N, 15.99; Found: C, 54.56; H, 6.50; N, 15.81.

*2-((4-(2,4-Dimethoxypyrimidin-5-yl)-1H-imidazol-1-yl)methoxy)ethyl pivalate (2-^t^BuAc).* The sugar **10** (0.30 g, 0.85 mmol) and 2,4-dimethoxy-5-(tributylstannyl)pyrimidine (0.44 g, 1.02 mmol) were used. The product was purified twice by flash column chromatography on silica (50–100% EtOAc in hexanes) to give the product as a light orange oil (0.16 g, 51%); ^1^H NMR (400 MHz, CDCl_3_) δ 9.05 (s, 1H), 7.95 (s, 1H), 7.53 (s, 1H), 5.42 (s, 2H), 4.22–4.23 (t, *J* = 4.7 Hz, 2H), 4.12 (s, 3H), 4.04 (s, 3H), 3.65–3.68 (t, *J* = 4.6 Hz, 2H), 1.18–1.20 (s, 9H); ^13^C NMR (101 MHz, CDCl_3_) δ 178.40, 167.03, 163.64, 154.57, 137.83, 133.21, 118.62, 108.77, 76.15, 66.55, 62.74, 54.04, 53.44, 38.30, 26.06; Elemental analysis calcd for C_17_H_24_N_4_O_5_ + 0.35 CH_3_OH + 0.05 H_2_O: C, 55.35; H, 6.83; N, 14.88; Found: C, 55.36; H, 6.75; N, 14.83.

*5-Iodo-4-methoxypyrimidine* (**12**). To a solution of 4-chloro-5-iodopyrimidine (0.5 g, 2.08 mmol) in anh. CH_3_OH (20 mL) under nitrogen was added NaOMe (30% by weight in CH_3_OH, 0.53 mL, 2.88 mmol) at room temperature. Then, the reaction was stirred at room temperature under nitrogen for 6 hours. Upon completion, the solvent was removed in vacuo to give a yellow solid. The crude mixture was purified by flash column chromatography on silica (0–50% EtOAc in hexanes) to give the product as a white solid (0.45 g, 92%); R_f_ = 0.86 (1:4 EtOAc: Hex); ^1^H NMR (500 MHz, CDCl_3_) δ 8.79 (s, 1H), 8.72 (s, 1H), 4.10 (s, 3H); ^13^C NMR (126 MHz, CDCl_3_) δ 167.1, 163.9, 157.4, 80.1, 54.8; MS (ESI+) *m/z* calcd for C_5_H_5_IN_2_O [M + H]^+^ 236.95, found: 236.56.

*4-Methoxy-5-(tributylstannyl)pyrimidine* (**13**). To a solution of **12** (0.89 g, 3.79 mmol) in anh. DMF (50 mL) under direct nitrogen bubble was added bis(tributyltin) (2.29 mL, 4.55 mmol) and Pd_2_dba_3_•CHCl_3_ (0.39 g, 0.38 mmol). Then, the reaction was stirred at 65 °C for 2.5 h. After completion, the reaction was cooled to room temperature, and the solvent was removed in vacuo to give a black oil. The crude mixture was resuspended in EtOAc (100 mL) and filtered over a celite pad. The filtrated was concentrated in vacuo to give a brown oil. Then, the crude product was purified by flash column chromatography on silica (0–70% EtOAc in hexanes) to give the pure product as a pale yellow oil (1.40 g, 93%); R_f_ = 0.64 (1:1 EtOAc: Hex); ^1^H NMR (500 MHz, CDCl_3_) δ 8.46 (s, 1H), 8.11 (s, 1H), 3.65 (s, 3H), 1.20–1.31 (m, 6H), 1.03–1.06 (m, 6H), 0.81–0.89 (m, 6H), 0.58–0.62 (m, 9H); ^13^C NMR (126 MHz, CDCl_3_) δ 188.5, 163.3, 158.5, 128.7, 53.1, 28.9, 27.3, 13.6, 9.6; MS (ESI+) *m/z* calcd for C_17_H_32_N_2_OSn [M + H]^+^ 401.15, found: 401.18.

*2-((4-(4-Methoxypyrimidin-5-yl)-1H-imidazol-1-yl)methoxy)ethan-1-ol* (**3**). The sugar **6** (0.35 g, 1.31 mmol) and stannyl pyrimidine **13** (0.63 g, 1.57 mmol) were used. The product was purified three times by flash column chromatography on silica (0–25% CH_3_OH in CH_2_Cl_2_) to give the product as a shiny white solid (0.135 g, 41%); ^1^H NMR (500 MHz, CD_3_OD) δ 9.05 (s, 1H), 8.62 (s, 1H), 7.97 (s, 1H), 7.83 (s, 1H), 5.51 (s, 2H), 4.16 (s, 3H), 3.67–3.69 (t, *J* = 5.0 Hz, 2H), 3.57–3.59 (t, *J* = 4.4 Hz, 2H); ^13^C NMR (126 MHz, CD_3_OD) δ 164.99, 154.93, 151.84, 138.32, 132.41, 120.82, 115.24, 76.54, 70.09, 60.51, 53.41; Elemental analysis calcd for C_11_H_14_N_4_O_3_: C, 52.79; H, 5.64; N, 22.39. Found: C, 52.62, H, 5.62, N, 22.17.

*2-((4-(4-Methoxypyrimidin-5-yl)-1H-imidazol-1-yl)methoxy)ethyl acetate (3-Ac).* The sugar **7** (0.35 g, 1.13 mmol) and stannyl pyrimidine **13** (0.54 g, 1.35 mmol) were used. The product was purified three times by flash column chromatography on silica (0–20% CH_3_OH in CH_2_Cl_2_) to give the product as a pale yellow-white solid (0.093 g, 30%); ^1^H NMR (500 MHz, CDCl_3_) δ 9.05 (s, 1H), 8.65 (s, 1H), 7.65 (s, 1H), 7.60 (s, 1H), 5.33 (s, 2H), 4.13–4.15 (t, *J* = 4.8 Hz, 2H), 4.06 (s, 3H), 3.58–3.60 (t, *J* = 4.6 Hz, 2H), 1.98 (s, 3H); ^13^C NMR (126 MHz, CDCl_3_) δ 170.89, 164.37, 155.43, 153.56, 137.72, 134.37, 121.32, 115.17, 76.55, 66.90, 62.88, 54.44, 21.43; Elemental analysis calcd for C_13_H_16_N_4_O_4_: C, 53.42; H, 5.52; N, 19.17. Found: C, 53.14, H, 5.56, N, 18.88.

*2-((4-(4-Methoxypyrimidin-5-yl)-1H-imidazol-1-yl)methoxy)ethyl propionate (3-EtAc).* The sugar **8** (0.35 g, 1.08 mmol) and stannyl pyrimidine **13** (0.52 g, 1.30 mmol) were used. The product was purified three times by flash column chromatography on silica (0–10% CH_3_OH in CH_2_Cl_2_) to give the product as a pale yellow oil (0.15 g, 45%); ^1^H NMR (400 MHz, CDCl_3_) δ 9.19 (s, 1H), 8.60 (s, 1H), 7.63 (s, 1H), 7.57 (s, 1H), 5.31 (s, 2H), 4.13–4.14 (t, *J* = 5.0 Hz, 2H), 4.05 (s, 3H), 3.57–3.59 (t, *J* = 4.6 Hz, 2H), 2.22–2.26 (m, 2H), 1.01–1.05 (t, *J* = 7.8 Hz, 3H); ^13^C NMR (126 MHz, CDCl_3_) δ 174.27, 164.74, 155.58, 153.57, 137.50, 134.17, 119.98, 115.11, 76.62, 66.79, 62.66, 54.05, 27.38, 9.03; Elemental analysis calcd for C_14_H_18_N_4_O_4_ + 0.1% CH3OH + 0.5% H_2_O: C, 53.17; H, 6.14; N, 17.59. Found: C, 52.99, H, 5.95, N, 17.40.

*2-((4-(4-Methoxypyrimidin-5-yl)-1H-imidazol-1-yl)methoxy)ethyl isobutyrate (3-^i^PrAc).* The sugar **9** (0.35 g, 1.04 mmol) and stannyl pyrimidine **13** (0.50 g, 1.24 mmol) were used. The product was purified three times by flash column chromatography on silica (first purification 40–100% EtOAc in hexanes, subsequent purifications 0–5% CH_3_OH in CH_2_Cl_2_) to give the product as a pale yellow oil (0.12 g, 37%); ^1^H NMR (400 MHz, CDCl_3_) δ 9.24 (s, 1H), 8.63 (s, 1H), 7.63 (s, 1H), 7.57 (s, 1H), 5.31 (s, 2H), 4.12–4.14 (t, *J* = 9.6 Hz, 2H), 4.04 (s, 3H), 3.57–3.58 (t, *J* = 5.0 Hz, 2H), 2.44–2.47 (m, 1H), 1.04–1.06 (d, *J* = 6.9 Hz, 6H); ^13^C NMR (126 MHz, CDCl_3_) δ 177.42, 165.12, 155.43, 153.66, 137.54, 134.37, 120.02, 115.26, 76.59, 66.34, 62.43, 54.04, 34.19, 19.99; Elemental analysis calcd for C_15_H_20_N_4_O_4_ + 0.3% H_2_O: C, 55.31; H, 6.37; N, 17.20. Found: C, 55.29, H, 6.33, N, 17.09.

*2-((4-(4-Methoxypyrimidin-5-yl)-1H-imidazol-1-yl)methoxy)ethyl pivalate (3-^t^BuAc). The* sugar **10** (0.38 g, 1.06 mmol) and stannyl pyrimidine **13** (0.51 g, 1.28 mmol) were used. The product was purified three times by flash column chromatography on silica (first purification 25–100% EtOAc in hexanes, subsequent purifications 0–5–10% CH_3_OH in CH_2_Cl_2_) to give the product as a clear oil (0.042 g, 12%); ^1^H NMR (500 MHz, CD_3_OD) δ 9.07 (s, 1H), 8.65 (s, 1H), 7.98 (s, 1H), 7.86 (s, 1H), 5.50 (s, 2H), 4.19–4.21 (m, 2H), 4.17 (s, 3H), 3.71–3.73 (m, 2H), 1.15 (s, 9H); ^13^C NMR (126 MHz, CD_3_OD) δ 178.41, 165.02, 155.01, 151.91, 138.36, 132.60, 120.77, 115.23, 76.21, 66.61, 62.73, 53.43, 38.30, 26.04; Elemental analysis calcd for C_16_H_22_N_4_O_4_ + 0.35 CH_3_OH + 0.1 H_2_O: C, 56.53; H, 6.85; N, 16.13; Found: C, 56.52; H, 6.83; N, 16.09.

*5-(Tributylstannyl)pyrimidin-2-amine* (**15**). To a solution of 2-amino-5-iodopyrimidine (0.50 g, 2.26 mmol) in anh. DMF (100 mL) under direct nitrogen bubbling was added bis(tributyltin) (1.37 mL, 2.71 mmol) and Pd_2_dba_3_•CHCl_3_ (0.23 g, 0.23 mmol). Then, the reaction was heated to 65 °C with stirring for 3 h under direct nitrogen bubbling. After completion, the solvent was removed in vacuo to give a black thick oil. The crude mixture was resuspended in EtOAc and filtered over a celite pad; then, the filtrate was concentrated in vacuo to give a dark liquid. Purification was performed using flash column chromatography on silica (0–100% EtOAc in hexanes) to give the pure product as a yellow oil (0.75 g, 86%); R_f_ = 0.76 (1:1 EtOAc: Hex); ^1^H NMR (500 MHz, CDCl_3_) δ 8.17 (s, 2H), 6.08 (br, 2H), 1.45–1.51 (m, 6H), 1.39–1.44 (m, 6H), 1.21–1.29 (m, 6H), 0.83–1.06 (m, 9H); ^13^C NMR (126 MHz, CDCl_3_) δ 164.32, 163.16, 119.22, 28.84, 27.44, 13.57, 8.09; MS (ESI+) *m/z* calcd for C_16_H_31_N_3_Sn [M + H]^+^ 386.15, found: 386.19.

*2-((4-(2-Aminopyrimidin-5-yl)-1H-imidazol-1-yl)methoxy)ethan-1-ol* (**4**). The sugar **6** (0.44 g, 1.63 mmol) and stannyl pyrimidine **15** (0.75 g, 1.95 mmol) were used. The product was purified four times by flash column chromatography on silica (0–30% CH_3_OH in CH_2_Cl_2_) to give the product as a pale pink solid (0.19 g, 50%); ^1^H NMR (500 MHz, CD_3_OD) δ 8.64 (s, 2H), 7.91 (s, 1H), 7.62 (s, 1H), 5.48 (s, 2H), 3.67–3.69 (t, *J* = 4.9 Hz, 2H), 3.56–3.58 (t, *J* = 4.6 Hz, 2H); ^13^C NMR (126 MHz, CD_3_OD) δ 163.2, 154.8, 138.5, 137.0, 117.6, 114.4, 77.4, 70.9, 60.2; Elemental analysis calcd for C_10_H_13_N_5_O_2_: C, 51.06; H, 5.57; N, 29.77. Found: C, 51.01, H, 5.47, N, 29.83.

*2-((4-(2-Aminopyrimidin-5-yl)-1H-imidazol-1-yl)methoxy)ethyl acetate* (**4-Ac**). The sugar **7** (0.48 g, 1.54 mmol) and stannyl pyrimidine **15** (0.71 g, 1.86 mmol) were used. The product was purified three times by flash column chromatography on silica (0–30% CH_3_OH in CH_2_Cl_2_) to give the product as a white solid (0.10 g, 24%); ^1^H NMR (400 MHz, CD_3_OD) δ 8.71 (s, 2H), 7.70 (s, 1H), 7.29 (s, 1H), 5.38 (s, 2H), 4.22–4.24 (t, *J* = 4.6 Hz, 2H), 3.66–3.68 (t, *J* = 4.6 Hz, 2H), 2.02 (s, 3H); ^13^C NMR (126 MHz, CD_3_OD) δ 171.5, 162.6, 154.6, 138.9, 136.8,117.2, 114.3, 75.6, 67.0, 63.1, 19.2; Elemental analysis calcd for C_12_H_15_N_5_O_3_ + 0.1 CH_3_OH + 0.05 CH_2_Cl_2_: C, 51.25; H, 5.49; N, 24.60. Found: C, 51.21, H, 5.47, N, 24.54.

*2-((4-(2-Aminopyrimidin-5-yl)-1H-imidazol-1-yl)methoxy)ethyl propionate* (**4-EtAc**). The sugar **8** (0.30 g, 0.93 mmol) and stannyl pyrimidine **15** (0.43 g, 1.11 mmol) were used. The product was purified four times by flash column chromatography on silica (0–20% CH_3_OH in CH_2_Cl_2_) to give the product as a white solid (0.09 g, 26%); ^1^H NMR (400 MHz, CD_3_OD) δ 8.60 (s, 2H), 7.87 (s, 1H), 7.57 (s, 1H), 5.42 (s, 2H), 4.15–4.17 (t, *J* = 4.6 Hz, 2H), 3.67–3.69 (t, *J* = 4.6 Hz, 2H), 2.24–2.28 (m, 2H), 1.02–1.06 (t, *J* = 7.8 Hz, 3H); ^13^C NMR (101 MHz, CD_3_OD) δ 174.58, 162.29, 154.81, 138.47, 136.81, 117.60, 114.45, 76.38, 66.75, 62.79, 26.78, 8.03; Elemental analysis calcd for C_13_H_17_N_5_O_3_ + 0.05 CH3OH + 0.95 H_2_O: C, 50.56 H, 6.21; N, 22.59. Found: C, 50.45, H, 6.07, N, 22.49.

*2-((4-(2-Aminopyrimidin-5-yl)-1H-imidazol-1-yl)methoxy)ethyl isobutyrate* (**4-^i^PrAc**). The sugar **9** (0.45 g, 1.33 mmol) and stannyl pyrimidine **15** (0.61 g, 1.60 mmol) were used. The product was purified four times by flash column chromatography on silica (0–20% CH_3_OH in CH_2_Cl_2_) to give the product as a pink solid (0.07 g, 17%); ^1^H NMR (400 MHz, CD_3_OD) δ 8.59 (s, 2H), 7.87 (s, 1H), 7.56 (s, 1H), 5.42 (s, 2H), 4.15–4.17 (t, *J* = 4.6 Hz, 2H), 3.67–3.69 (t, *J* = 4.6 Hz, 2H), 2.46–2.50 (m, 1H), 1.06–1.08 (d, *J* = 6.8 Hz, 6H); ^13^C NMR (101 MHz, CD_3_OD) δ 177.42, 162.32, 155.05, 138.65, 136.98, 117.69, 114.61, 76.41, 66.72, 62.80, 33.55, 18.45; Elemental analysis calcd for C_14_H_19_N_5_O_3_: C, 55.07 H, 6.27; N, 22.94. Found: C, 55.02, H, 6.13, N, 22.73.

*2-((4-(2-Aminopyrimidin-5-yl)-1H-imidazol-1-yl)methoxy)ethyl pivalate* (**4-^t^BuAc**). The sugar **10** (0.30 g, 0.85 mmol) and stannyl pyrimidine **15** (0.39 g, 1.02 mmol) were used. The product was purified twice by flash column chromatography on silica (0–20% CH_3_OH in CH_2_Cl_2_) to give the product as a white solid (0.06 g, 20%); ^1^H NMR (400 MHz, CDCl_3_) δ 8.63 (s, 2H), 7.63 (s, 1H), 7.22 (s, 1H), 5.48 (br, 2H), 5.30 (s, 2H), 4.15–4.16 (t, *J* = 3.7 Hz, 2H), 3.58–3.60 (t, *J* = 3.7 Hz, 2H), 1.09–1.11 (s, 9H); ^13^C NMR (101 MHz, CDCl_3_) δ 178.40, 162.17, 155.12, 138.17, 137.85, 118.49, 113.33, 76.58, 66.82, 62.64, 38.78, 27.17; Elemental analysis calcd for C_15_H_21_N_5_O_3_: C, 56.41; H, 6.63; N, 21.93. Found: C, 56.11, H, 6.47, N, 21.65.

*5-Iodopyrimidin-4-amine* (**17**).^14^ To a solution of 4-aminopyrimidine (0.80 g, 8.5 mmol) in glacial AcOH (20 mL) under nitrogen was added N-iodosuccinimide (2.30 g, 10.20 mmol) with stirring. The reaction refluxed at 80 °C for two hours. Upon completion, the reaction was cooled to room temperature, and the solvent was removed in vacuo. The resulting yellow solid was resuspended in EtOAc, quenched with NaHCO_3_ (50 mL), washed with Na_2_S_2_O_3_ (50 mL), and the organic layer was dried with MgSO_4_ and gravity filtered to give a yellow solid. Then, the crude product was purified by flash column chromatography on silica (0–10% CH_3_OH in CH_2_Cl_2_) to give the pure product as a pale yellow solid (1.5 g, 80%); R_f_ = 0.48 (1:1 EtOAc: Hex); Spectral data is in agreement with literature values.^14^

*5-(tributylstannyl)pyrimidin-4-amine* (**18**).^14^ To a solution of **17** (0.75 g, 3.39 mmol) in anh. DMF (50 mL) under direct bubbling nitrogen was added bis(tributyl)tin (2.05 mL, 4.07 mmol) and Pd_2_dba_3_•CHCl_3_ (0.35 g, 0.34 mmol) with stirring. Then, the reaction was heated to 65 °C for 2.5 h. Upon completion, the reaction was cooled to room temperature and the solvent was removed in vacuo. Then, the resulting oil was resuspended in EtOAc, filtered over a celite pad, and the filtrate was concentrated in vacuo to give a yellow oil. Then, the crude product was purified by flash column chromatography on silica (10 -60% EtOAc in hexanes) to give the pure product as a pale yellow solid (0.80 g, 61%); R_f_ = 0.65 (1:1 EtOAc: Hex); Spectral data is in agreement with literature values.^14^

*2-((4-(4-Aminopyrimidin-5-yl)-1H-imidazol-1-yl)methoxy)ethan-1-ol* (**5**). The sugar **6** (0.25 g, 0.93 mmol) and stannyl pyrimidine **18** (0.43 g, 1.12 mmol) were used. The product was purified five times by flash column chromatography on silica (0–15% CH_3_OH in CH_2_Cl_2_) to give the product as a pale pink solid (0.03 g, 11%); ^1^H NMR (500 MHz, CD_3_OD) δ 8.44 (s, 1H), 8.29 (s, 1H), 7.93 (s, 1H), 7.78 (s, 1H), 5.50 (s, 2H), 3.67–3.69 (m, 2H), 3.56–3.58 (m, 2H); ^13^C NMR (126 MHz, CD_3_OD) δ 160.25, 155.37, 150.03, 137.43, 136.56, 116.33, 110.63, 76.53, 70.11, 60.50. Elemental analysis calcd for C_10_H_13_N_5_O_2_ + 0.05 CH_2_Cl_2_: C, 50.40; H, 5.41; N, 29.24; Found: C, 50.55; H, 5.47; N, 29.10.

*2-((4-(4-Aminopyrimidin-5-yl)-1H-imidazol-1-yl)methoxy)ethyl acetate* (**5-Ac**). The sugar **7** (0.27 g, 0.87 mmol) and stannyl pyrimidine **18** (0.40 g, 1.04 mmol) were used. The product was purified five times by flash column chromatography on silica (0–15% CH_3_OH in CH_2_Cl_2_) to give the product as a pink solid (0.03 g, 11%); ^1^H NMR (500 MHz, DMSO-d_6_) δ 8.52 (s, 1H), 8.26 (s, 1H), 8.00 (s, 1H), 7.91 (s, 1H), 5.45 (s, 2H), 4.10–4.12 (t, *J* = 4.7 Hz, 2H), 3.65–3.67 (t, *J* = 4.7 Hz, 2H), 2.01 (s, 3H); ^13^C NMR (125 MHz, CDCl_3_) δ 171.13, 159.92, 156.76, 151.23, 138.36, 133.76, 114.90, 109.90, 76.58, 66.57, 62.71, 20.42; Elemental analysis calcd for C_12_H_15_N_5_O_3_ + 0.55 CH_2_Cl_2_: C, 46.52; H, 5.01; N, 21.62; Found: C, 46.42; H, 4.87; N, 21.60.

*2-((4-(4-Aminopyrimidin-5-yl)-1H-imidazol-1-yl)methoxy)ethyl propionate* (**5-EtAc**). The sugar **8** (0.27 g, 0.84 mmol) and stannyl pyrimidine **18** (0.39 g, 1.01 mmol) were used. The product was purified four times by flash column chromatography on silica (0–10% CH_3_OH in CH_2_Cl_2_) to give the product as a clear oil (0.015 g, 6.1%); ^1^H NMR (500 MHz, CD_3_OD) δ 8.45 (s, 1H), 8.29 (s,1H), 7.93 (s, 1H), 7.79 (s, 1H), 5.49 (s, 2H), 4.19–4.21 (m, 2H), 3.72–3.74 (m, 2H), 2.29–2.34 (q, *J* = 7.6 Hz, 2H), 1.06–1.09 (t, *J* = 7.6 Hz, 3H); ^13^C NMR (126 MHz, CD_3_OD) δ 174.53, 160.25, 155.41, 150.04, 137.43, 136.69, 116.28, 110.56, 76.31, 66.73, 62.71, 26.70, 7.93; Elemental analysis cald for C_13_H_17_N_5_O_3_ + 0.1 CH_3_OH + 0.4 H_2_O: C, 52.12; H, 6.08; N, 23.21; Found: C, 52.26; H, 5.82; N, 23.03.

*2-((4-(4-Aminopyrimidin-5-yl)-1H-imidazol-1-yl)methoxy)ethyl isobutyrate* (**5-^i^PrAc**). A solution of **17** (0.2 g, 0.91 mmol) in anh. THF (20 mL) was cooled to −78 °C in an acetone/CO_2(s)_ bath under nitrogen. EtMgBr (0.33 mL, 1.0 mmol) was added dropwise and the reaction was stirred for two minutes, followed by the addition of TMSCl (0.14 mL, 1.09 mmol), after which the reaction was stirred for 5 min. Another addition of EtMgBr (0.33 mL, 1.0 mmol) was added dropwise, and the reaction was stirred for 2 min; then, another addition of TMSCl (0.14 mL, 1.09 mmol) was added, and the reaction was stirred for 5 min. A final addition of EtMgBr (0.33 mL, 1.0 mmol) was added, and the reaction stirred for 10 min; then, ZnCl_2_ (1.81 mL, 1.81 mmol) was added dropwise. The solution was stirred at −78 °C for 10 min, and then warmed to room temperature and stirred for 2 h. The organozinc mixture was then added dropwise to a solution of **9** (0.34 g, 1.0 mmol), Pd(PPh_3_)_4_ (0.11 g, 0.09 mmol), and CuI (0.01 g, 0.05 mmol) in anh. THF (10 mL). Then, the reaction stirred under nitrogen for 18 hours at room temperature. Upon completion, the reaction was quenched with saturated ethylenediaminetetraacetic acid (EDTA) (10 mL), after which it was concentrated in vacuo, extracted three times with CH_2_Cl_2_, washed with brine, and the organics were dried with MgSO_4_. After gravity filtration, the solvent was removed in vacuo to give a dark oil. Then, the crude mixture was purified twice via flash column chromatography on silica (0–10% CH_3_OH (10% NH_4_OH) in DCM) to give the product as a pink oil (0.16 g, 48%); ^1^H NMR (500 MHz, CD_3_OD) δ 8.45 (s, 1H), 8.29 (s, 1H), 7.94 (s, 1H), 7.80 (s, 1H), 5.50 (s, 2H), 4.20–4.21 (m, 2H), 3.72–3.74 (m, 2H), 2.52–2.53 (m, 1H), 1.11–1.13 (d, *J* = 7.0 Hz, 6H); ^13^C NMR (126 MHz, (CD_3_)_3_CO) δ 176.26, 160.13, 156.41, 151.08, 137.78, 137.24, 115.69, 109.97, 76.43, 66.84, 62.68, 33.54, 18.42; Elemental analysis calcd for C_14_H_19_N_5_O_3_ + 0.75 H_2_O: C, 52.74; H, 6.48; N, 21.96; Found: C, 52.55; H, 6.21; N, 21.82.

*2-((4-(4-Aminopyrimidin-5-yl)-1H-imidazol-1-yl)methoxy)ethyl pivalate* (**5-^t^BuAc**). A solution of **17** (0.2 g, 0.90 mmol) in anh. THF (15 mL) was cooled to −78 °C in an acetone/CO_2(s)_ bath under nitrogen. EtMgBr (0.33 mL, 1.0 mmol) was added dropwise, and the reaction was stirred for two minutes, followed by the addition of TMSCl (0.14 mL, 1.09 mmol) and the reaction was stirred for another 5 min. Another addition of EtMgBr (0.33 mL, 1.0 mmol) was added dropwise, after which the reaction stirred for 2 min; then, another addition of TMSCl (0.14 mL, 1.09 mmol) was added, and the reaction was stirred for 5 min. A final addition of EtMgBr (0.33 mL, 1.0 mmol) was added, and the reaction stirred for 10 min; then, ZnCl_2_ (1.81 mL, 1.81 mmol) was added dropwise. The solution was stirred at −78 °C for 10 min, and then warmed to room temperature and stirred for 2 h. Then, the organozinc mixture was added dropwise to a solution of **10** (0.35 g, 1.0 mmol), Pd(PPh_3_)_4_ (0.01 g, 0.09 mmol), and CuI (0.01 g, 0.04 mmol) in anh. THF (10 mL). Then, the reaction was stirred under nitrogen for 18 hours at room temperature. Upon completion, the reaction was quenched with saturated EDTA (10 mL), after which it was concentrated in vacuo, extracted three times with CH_2_Cl_2_, washed with brine, and the organics were dried with MgSO_4_. After gravity filtration, the solvent was removed in vacuo to give a dark oil. Then, the crude mixture was purified twice via flash column chromatography on silica (0–10% CH_3_OH (10% NH_4_OH) in DCM) to give the product as a light pink oil (0.03, 9.4%); ^1^H NMR (500 MHz, CD_3_OD) δ 8.54 (s, 1H), 8.30 (s, 1H), 7.98 (s, 1H), 7.87 (s, 1H), 5.58 (s, 2H), 4.19–4.21 (m, 2H), 3.72–3.78 (m, 2H), 1.16 (s, 9H); ^13^C NMR (126 MHz, CDCl_3_) δ 179.09, 159.91, 156.79, 151.26, 138.42, 136.21, 114.81, 109.90, 76.66, 66.93, 62.52, 38.74, 27.71; Elemental analysis calcd for C_14_H_19_N_5_O_3_ + 0.05 CH_3_OH + 0.8 H_2_O: C, 54.01; H, 6.53; N, 20.50. 

### 4.3. Biological Assays

All work with infectious virus was conducted in a Biological Safety Level 4 (BSL-4) laboratory at the Centers for Disease Control and Prevention. Laboratorians adhered to international practices appropriate for this biosafety level.

Huh7 cells were seeded at 4 × 10^3^ cells/well (384-well plates) in FluoroBrite Dulbecco’s modified Eagle’s medium (ThermoFisher, Waltham, MA, USA), supplemented with 10% (*v*/*v*) fetal calf serum (Hyclone, GE Healthcare, Chicago, IL, USA) and 1x penicillin and streptomycin (ThermoFisher). The following day, compounds were added using an Echo 550 acoustic liquid handler (Labcyte, San Jose, CA, USA), and the plates were transferred to a BSL-4 laboratory. Cells were infected with EBOV-ZsGreen reporter virus [23,24] 2–3 h after the addition of compounds. ZsGreen fluorescence was determined 72 h post-infection using a Synergy H1MD plate reader (BioTek, Winooski, VT, USA). Cell viability was determined at the same time on compound-treated, mock-infected cells using CellTiter-Glo (Promega, Madison, WI). GraphPad Prism (GraphPad Software, La Jolla, CA, USA) was used to fit a four-parameter equation to semilog plots of the concentration–response data and to derive the concentration of compound that inhibited 50% of the virus-induced cell death (EC_50_).

For statistical analysis, EC_50_ values from repeat experiments were compared using the Mann–Whitney test in GraphPad Prism. Statistical significance was assumed with a *p* value < 0.05.

### 4.4. NMR Conformational Analysis

^1^H NMR spectra were recorded on an Agilent Technologies DD2 300 NMR spectrometer at the Slovenian NMR center at 297.8 MHz. Spectra were acquired at 3-mM concentration in 1% DMSO-d_6_/D_2_O. Gradient-based 1D NOESY pulse sequence was used for the selective inversion and assessment of conformational features of fleximers based on observed NOE enhancements.

## 5. Conclusions

In this study, an SAR was pursued in order to increase antiviral activity against EBOV and decrease cytotoxicity. While none of the analogues analyzed were cytotoxic up to 100 µM, none of the analogues were more active against EBOV than compound **1**. Regardless, it was determined that the 4-methoxy series were overall more active than the 2-amino series, suggesting that the 4-methoxy moiety may be more important for activity. Furthermore, compounds with logP values greater than 1 but less than 2.4 offer the necessary lipophilicity for activity. It was also found that compounds within series **1** and **2** adopt the typical anti-conformation, whereas compounds within series **5** adopt a syn conformation, thus offering another possible explanation for the differences in antiviral activity. With these initial results in hand, the synthesis of additional analogues, especially those with a methoxy group at the 4 position will be considered, in order to continue to try and further explore antiviral activity. The results of those studies will be reported elsewhere as they become available. 

## Figures and Tables

**Table 1:** Antiviral activity of series **1–5** against Ebola virus (EBOV) in Huh7 cells.

R Group	1	2	3	4	5
	^a^EC_50_ (µM)	EC_50_ (µM)	EC_50_ (µM)	EC_50_ (µM)	EC_50_ (µM)
**H**	1.49 ± 0.98	23.44 ± 20.28	78.88 ± 17.14	>100	>100
**Ac**	>100	16.34 ± 11.46	35.36 ± 20.37	52.89 ± 28.25	>100
**EtAc**	62.18 ± 18.99	>100	51.87 ± 19.58	>100	>100
**^i^PrAc**	>100	31.96 ± 9.12	38.29 ± 28.15	53.99 ± 23.74	>100
**^t^BuAc**	7.21 ± 1.85	81.34 ±10.15	51.40 ± 10.01	34.42 ± 20.01	72.29 ± 18.74

^a^EC_50_: effective concentration showing 50% inhibition of virus-expressed ZsGreen fluorescence.; all compounds were nontoxic up to 100 µM in uninfected cells.

**Table 2:** Lipophilicity (logP) values of series **1**–**5**.

R Group	Series (logP)				
	1	2	3	4	5
**H**	0.02	0.7	0.31	−0.57	−0.36
**Ac**	0.25	0.93	0.54	−0.34	−0.14
**EtAc**	0.9	1.58	1.2	0.31	0.52
**^i^PrAc**	1.47	2.15	1.76	0.88	1.09
**^t^BuAc**	2.18	2.85	2.47	1.59	1.79

## References

[1] Seley-Radtke K.L., Yates M.K. (2018). The evolution of nucleoside analogue antivirals: A review for chemists and non-chemists. Part 1: Early structural modifications to the nucleoside scaffold. Antiviral Res..

[2] Yates M.K., Seley-Radtke K.L. (2019). The evolution of antiviral nucleoside analogues: A review for chemists and non-chemists. Part II: Complex modifications to the nucleoside scaffold. Antiviral Res..

[3] Seley K.L., Zhang L., Hagos A. (2001). “Fleximers”. Design and synthesis of two novel split nucleosides. Org. Lett..

[4] Seley K.L., Zhang L., Hagos A., Quirk S. (2002). “Fleximers”. Design and synthesis of a new class of novel shape-modified nucleosides^1^. J. Org. Chem..

[5] Seley K.L., Quirk S., Salim S., Zhang L., Hagos A. (2003). Unexpected inhibition of S-adenosyl-L-homocysteine hydrolase by a guanosine nucleoside. Bioorg. Med. Chem. Lett..

[6] Quirk S., Seley K.L. (2005). Identification of catalytic amino acids in the human GTP fucose pyrophosphorylase active site. Biochemistry.

[7] Seley K.L., Salim S., Zhang L., O’Daniel P.I. (2005). “Molecular chameleons”. Design and synthesis of a second series of flexible nucleosides. J. Org. Chem..

[8] Quirk S., Seley K.L. (2005). Substrate discrimination by the human GTP fucose pyrophosphorylase. Biochemistry.

[9] Zhang Z., Wauchope O.R., Seley-Radtke K.L. (2008). Mechanistic studies in the synthesis of a series of thieno-expanded xanthosine and guanosine nucleosides. Tetrahedron.

[10] O’Daniel P.I., Jefferson M., Wiest O., Seley-Radtke K.L. (2008). A computational study of expanded heterocyclic nucleosides in DNA. J. Biomol. Struct. Dyn..

[11] Wauchope O.R., Tomney M.J., Pepper J.L., Korba B.E., Seley-Radtke K.L. (2010). Tricyclic 2′-C-Modified Nucleosides as Potential Anti-HCV Therapeutics. Org. Lett..

[12] Zimmermann S.C., Sadler J.M., Andrei G., Snoeck R., Balzarini J., Seley-Radtke K.L. (2011). Carbocyclic 5′-nor “reverse” fleximers. Design, synthesis, and preliminary biological activity. Med. Chem. Comm..

[13] Wauchope O.R., Johnson C., Krishnamoorthy P., Andrei G., Snoeck R., Balzarini J., Seley-Radtke K.L. (2012). Synthesis and biological evaluation of a series of thieno-expanded tricyclic purine 2’-deoxy nucleoside analogues. Bioorg. Med. Chem..

[14] Wauchope O.R., Velasquez M., Seley-Radtke K. (2012). Synthetic Routes to a Series of Proximal and Distal 2′-Deoxy Fleximers. Synthesis (Stuttg).

[15] Zimmermann S.C., Sadler J.M., O’Daniel P.I., Kim N.T., Seley-Radtke K.L. (2013). “Reverse” carbocyclic fleximers: synthesis of a new class of adenosine deaminase inhibitors. Nucleosides Nucleotides Nucleic Acids.

[16] Zimmermann S.C., O’Neill E., Ebiloma G.U., Wallace L.J., De Koning H.P., Seley-Radtke K.L. (2014). Design and synthesis of a series of truncated neplanocin fleximers. Molecules.

[17] Peters H.L., Jochmans D., de Wilde A.H., Posthuma C.C., Snijder E.J., Neyts J., Seley-Radtke K.L. (2015). Design, synthesis and evaluation of a series of acyclic fleximer nucleoside analogues with anti-coronavirus activity. Bioorg. Med. Chem. Lett..

[18] Chen Z., Jochmans D., Ku T., Paeshuyse J., Neyts J., Seley-Radtke K.L. (2015). Bicyclic and Tricyclic "Expanded" Nucleobase Analogues of Sofosbuvir: New Scaffolds for Hepatitis C Therapies. ACS Infect. Dis..

[19] Chen Z., Ku T.C., Seley-Radtke K.L. (2015). Thiophene-expanded guanosine analogues of Gemcitabine. Bioorg. Med. Chem. Lett..

[20] Yates M.K., Raje M.R., Chatterjee P., Spiropoulou C.F., Bavari S., Flint M., Soloveva V., Seley-Radtke K.L. (2017). Flex-nucleoside analogues-Novel therapeutics against filoviruses. Bioorg. Med. Chem. Lett..

[21] Polak M., Seley K.L., Plavec J. (2004). Conformational properties of shape modified nucleosides--fleximers. J. Am. Chem. Soc..

[22] Ku T., Lopresti N., Shirley M., Mori M., Marchant J., Heng X., Botta M., Summers M.F., Seley-Radtke K.L. (2019). Synthesis of distal and proximal fleximer base analogues and evaluation in the nucleocapsid protein of HIV-1. Bioorg. Med. Chem..

[23] Welch S.R., Guerrero L.W., Chakrabarti A.K., McMullan L.K., Flint M., Bluemling G.R., Painter G.R., Nichol S.T., Spiropoulou C.F., Albariño C.G. (2016). Lassa and Ebola virus inhibitors identified using minigenome and recombinant virus reporter systems. Antiviral Res..

[24] Albariño C.G., Wiggleton Guerrero L., Lo M.K., Nichol S.T., Towner J.S. (2015). Development of a reverse genetics system to generate a recombinant Ebola virus Makona expressing a green fluorescent protein. Virology.

